# Liverpool Babies

**Published:** 1924-11

**Authors:** 


### LIVERPOOL BABIES.

They are hammering away at their infantile mortality rate in Liverpool. As in other parts of the country, there is the gratifying decline of about 50 per cent. in the rate since the beginning of the century, but the reduced figure of 99 for 1923 compares unfavourably with that of most areas, and invites continuous attack. Dr. Hope, the Medical Officer for the city, observes in his report for 1923 that it is unfortunate that a corresponding reduction cannot be recorded in the case of the mothers:—

> No doubt the problems surrounding maternity are more difficult to solve than those relating to the lives of infants, but closer attention is being paid to the dangers to which the mothers are subject and which at the present time are not far removed from those of twenty-five years ago. A highly important step, however, has been made in providing Maternity Homes and Ante-natal and Post-natal Clinics, as it has been demonstrated that a large proportion of the accidents which occur during pregnancy and child-birth can be successfully forestalled and prevented if the patient is under medical supervision previous to her confinement. Means can also be taken at the post-natal Clinics to assist in restoring the mother to her normal health. The expansion of these arrangements, which are now becoming widely known and extensively adopted, should assist materially in reducing the preventable deaths of women during child-birth, and reduce not only the preventable deaths, but also the sickness amongst women caused by unskilful or neglectful midwifery.

A notable step forward in the case of the Liverpool babies is recorded:—The Carnegie Welfare Centre was opened on behalf of the Trustees of the Carnegie United Kingdom Trust, the donors of the building, by Miss Haldane, one of the Trustees The building, which is conveniently situated close to the University School of Hygiene, the Royal Liverpool Children's Hospital and the new Maternity Hospital, will provide accommodation for Pre-Maternity and Infant Welfare Clinics, together with certain auxiliary work, such as classes for mothers in cookery, sewing, and housework. In the upper floors of the building there are observation wards, with 12-15 cots, to which infants who are suffering from minor dietetic troubles can be admitted and kept under observation and treatment. The building is well equipped, and will be a most valuable addition to the Maternity and Child Welfare Institutions of the City.

